# Iron oxide nanoparticles coated with bioactive materials: a viable theragnostic strategy to improve osteosarcoma treatment

**DOI:** 10.1186/s11671-024-04163-w

**Published:** 2025-01-30

**Authors:** Amy Sarah Benjamin, Sunita Nayak

**Affiliations:** 1 School of Advanced Sciences, Vellore Institute of Technology, Vellore, Tamil Nadu 632014 India; 2 School of Biosciences and Technology, Vellore Institute of Technology, Vellore, Tamil Nadu 632014 India

**Keywords:** Nanotechnology, Iron oxide nanoparticles, Magnetic properties, Bio-active polymers, Osteosarcoma, Cancer nanomedicine

## Abstract

Osteosarcoma (OS) is distinguished as a high-grade malignant tumor, characterized by rapid systemic metastasis, particularly to the lungs, resulting in very low survival rates. Understanding the complexities of tumor development and mutation is the need of the hour for the advancement of targeted therapies in cancer care. A significant innovation in this area is the use of nanotechnology, specifically nanoparticles, to tackle various challenges in cancer treatment. Iron oxide nanoparticles stand out in both therapeutic and diagnostic applications, offering a versatile platform for targeted drug delivery, hyperthermia, magneto-thermal therapy, and combinational therapy using modulation of ferroptosis pathways. These nanoparticles are easy to synthesize, non-toxic, biocompatible, and display enhanced circulation time within the system. They can also be easily conjugated to anti-cancer drugs, targeting agents, or genetic vectors that respond to specific stimuli or pH changes. The surface functionalization of these nanoparticles using bioactive molecules unveils a promising and effective nanoparticle system for assisting osteosarcoma therapy. This review will summarize the current conventional therapies for osteosarcoma and their disadvantages, the synthesis and modification of iron oxide nanoparticles documented in the literature, cellular targeting and uptake mechanism, with focus on their functionalization using natural biomaterials and application strategies towards management of osteosarcoma. The review also compiles the translational challenges and future prospects that must be addressed for clinical advancements of iron oxide based osteosarcoma treatment in the future.

**Graphical abstract:**

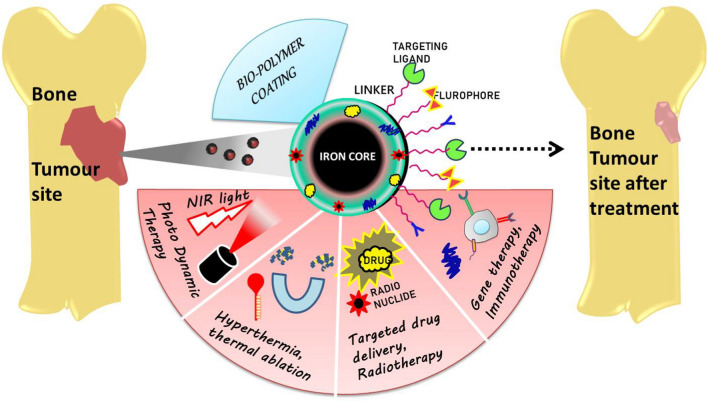

## Introduction

Cancer is one among the major global health problems affecting millions of the population each year. According to World Health Organization, the mortality and incidence data for 2020 was recorded as 10 million deaths due to cancer [1] while in India, according to the consolidated report by the National Cancer Registry programme for the year 2020 the estimated number of people affected with cancer are around 2.7 million estimating 1 in 9 people to have the risk of developing cancer before the age of 75 [2]. Bone tumor constitutes only 1% of the overall cancer diagnosis but with rapid aggressive cancer spread, high mortality rate, concurrent recurrence, high disability and low 5-year survival rate it poses a lethal threat to the human population. Osteosarcoma (OS) is one among the most prevalent primary malignant bone tumors affecting children and teenagers, with an occurrence of more than 3.4 per million in a year [3]most often located at the metaphysis of long bones, in the distal femur, tibia and humerus also found in the diaphysis in some cases. The clinical symptoms of bone tumor initiate with severe pain, swelling of the affected bone site, hypercalcemia and pathological fracture [4].The other common bone tumors differing based on their bone tissue origin are chondrosarcoma, Ewing tumor and chordomas; the prognosis for patients with bone tumor depends on many factors including the type, location of tumor, stage of growth of the tumor tissue, metastasis position during diagnosis, tertiary health conditions of the person and response to medication [5–7].The conventional treatment in the current clinical scenario comprises of surgical resection accompanied adjuvant therapy with chemotherapy, radiotherapy or targeted therapy; in an aggressive metastatic state or condition, amputation of the diseased site [8]. The complication and heterogeneity of bone tumors calls for a better understanding to improve therapeutic strategies, to reduce recurrence, and improve the survival condition after treatment.

The different FDA approved drugs used in the chemotherapy health care systems are paclitaxel, Adriamycin, cisplatin, Denosumab, doxorubicin which have been crucially used for chemotherapeutic management of the disease which is the only solution despite the facts of its damage to normal cells, multidrug resistance and effective tumor targeting [9]. Accompanying the conventional therapies there are also emerging therapies like immunotherapy [10], PDT (Photo Dynamic Therapy) [11], PTT (Photo thermal therapy) [12], MHT (Magnetic hyperthermia therapy) [13], Gene therapy [14] and CDT (Chemo dynamic therapy) [15] which are being explored towards cancer for a much more efficient solution [16]. The emergence of nanotechnology has given a ray of hope resulting to bridge the plentiful clinical challenges faced with conventional therapies. The use of inorganic magnetic nanomaterials in therapeutic and diagnostic applications as drug delivery systems, targeting and contrast agents, to reduce side effects and improve the treatment efficiency for tumors has been researched extensively and during the past decades. Progressively these magnetic nanomaterials show advantages over the conventional treatment strategies. One of the remarkable study of iron oxide nanoparticles(IONPs) which are also called super paramagnetic iron oxide nanoparticles (SPIONS) for cancer treatment has emerged as an efficient representative, identified to have potential features particularly to target cancer cells and provide an external stimuli without damage to cells with commendable contribution in precision medicine [17]. Its applications has been well explored over the recent years with a subsequent interest towards the field of cancer research for targeted drug delivery, gene targeting, hyperthermia, and magnetic resonance imaging. [18]. Since they are non-toxic to cells and possess the ability to exhibit super-paramagnetism property it makes them very attractive to researchers to target the cancer cells from a magnetic perspective externally which could open new doors to non-invasive procedures. A few other prominent areas where iron oxide nanoparticles are effectively being used;cell based study like cell labeling, as a tool for cell biology research to separate and purify cell populations; magnetic field- guided carriers for drug delivery; magnetic hyperthermia; and magneto-fection [19]. Given their strong magnetization in an external magnetic field and noticeable T2/T2* relaxation, super paramagnetic iron oxide nanoparticles, or SPIONs, function as effective contrast agents for magnetic resonance imaging (MRI) [20].Commercially, apart from cancer research they have been tailored to administer to various fields like opto-electronics, enhanced automation and robotics, chemical catalysts, oil and gas industries, textiles, nano-electronics, tissue regeneration and also pharmaceutical industry [21]. The use of these iron oxide nanomaterials in their bare state may result in mild aggregation due to their inherent magnetic property; however, to overcome this limitation, they are coated with various organic surfactants, biomolecules, non-synthetic and synthetic polymers, polysaccharides, and biomaterials respective to the application, to improve dispersiblity, colloidal stability, efficiency and multiply their benefits.

Iron oxide based nanosystems modified using biomaterials obtained from natural sources of origin are among the most favorable for precision medicine in the current medical scenario as they are based on natural genesis such as sugars, proteins, polysaccharides, peptides, nucleic acids derived from plants, animals or other biological resources, displaying a much improvised scope to be translated within the body system [22] with a greater potential significance. Several studies on iron oxide nanoparticles for smart targeting techniques, efficient tumor accumulation, and controlled release of drug payload in breast, head and neck, skin, and other cancers have been reported so far [7]. Nanoparticles have poor penetration into osteosarcoma tissues due to its strong extracellular matrix, thus increasing the penetration effect using various factors is essential. The efficacy of functionalized iron oxide nanoparticles against osteosarcoma is a confined direction to summarize the published and new research on the potential of biomaterial coated iron oxide nanoparticles for osteosarcoma treatment. The emphasis of iron oxide nanoparticles for osteosarcoma has been compiled to provide an easy and informative detail for progress in this direction of nanomedicine. This review aims to discuss the types of bone tumors the current clinical treatment plans employed for the disease management, highlighting the significance of nanotechnology based advancements using iron oxide nanoparticles. The various synthesis methods, surface modification, functionalization, effect of toxicity, targeting strategies has been discussed with significance of biomaterial modified nanoparticles and their application. Moreover, the effect on cancer cell lines, animal models and clinical trials in progress have been discussed. Finally, the iron oxide nanoparticles modified using natural biomaterials towards the treatment of osteosarcoma, its challenges, limitations and future prospects for the progress in cancer theranostics.

## Osteosarcoma: etiology, diagnosis, conventional and upcoming clinical treatment strategies

Osteosarcoma being the major cancer type of the bone affects a major population of both children and adults, progresses in extreme metastases of the lungs [7, 23]. The etiology of OS most often occurs at the metaphysis of long bones but is also found in the diaphysis in some cases [24] and is still being studied extensively for a clear discretion of the disease condition. The histopathological subtypes of osteosarcoma are divided based on their cellular components. In Telangiectatic osteosarcoma (TOS) which is about 4% of the overall OS rate is characterized by blood filled cavities and dense cells on the lining and outer rim of the bone with the bone eaten or destructed bone seen in an X-Ray image [25]. Small–cell osteosarcoma which contributes to around 2% have spherical hypo-chromatic nuclei with visible nuclear polymorphism resembles Ewing’s sarcoma but differs in the production of osteoid by tumor cells [26, 27]. Low-grade osteosarcoma affects after the age 30 with the same features of parosteal sarcoma and can be little difficult to recognize [28, 29]. Parosteal osteosarcoma originates from the periostem and affects the posterior part of the distal femur along with the tibia and humerus with characteristic features of a dense lobed mass avoiding the medullary cavity seen in radiograph [30, 31]. The other surface based types are periosteal which is cartilaginous matrix seen in the histopathological examination and X-Ray image [32].

**Fig. 1:**
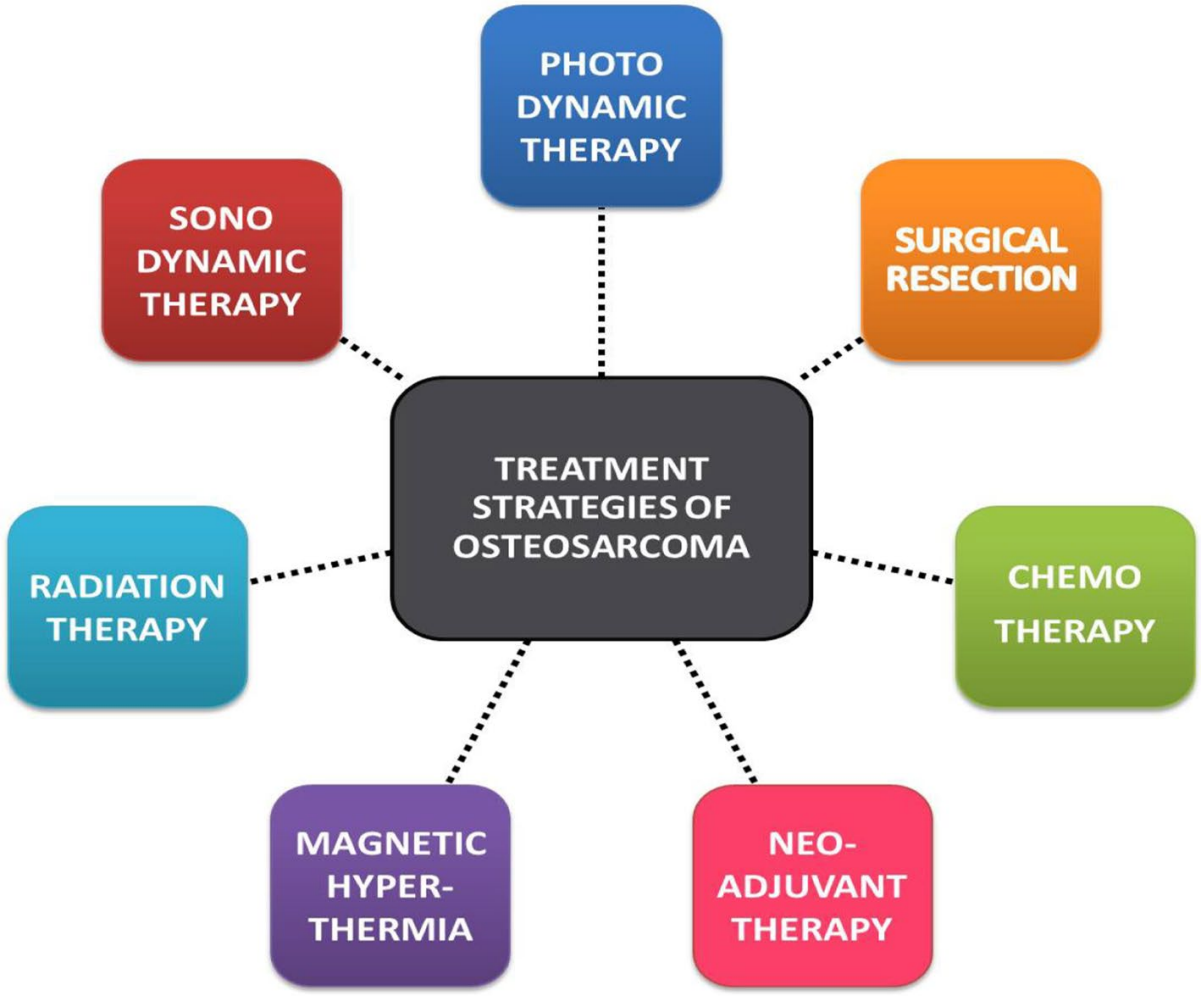
Present treatment strategies of osteosarcoma in the clinical scenario [10]

The most common symptoms include fractures, compression of the spinal cord, pain, spinal instability and brittleness of the bone. Diagnosis of the cancerous tissue involves radiography images of the bone site with the adjacent joint to view the irregular lesion, [33, 34] accompanied by MRI (magnetic resonance imaging) to examine the amount of the lesion’s invasion into the soft tissue, its extension to the neighboring tissues, its neurovascular structures and bone marrow infection [35, 36]. Computed tomography (CT) is useful to observe the mineralization of the bone, fracture details, tumor mass dimensions and neurovascular invasions along with bone scintigraphy. Angiography is done to view the anatomy for planning operation in certain cases of common anatomical anomalies [37], PET scans are also being used to evaluate the primary and metastatic lesions from bone to lungs and also to assess the response of chemotherapy to the cancerous cells. A biopsy is for the definitive diagnosis carried out very carefully by a radiologist or surgeon with or without imaging assistance depending on the severity, in severe conditions imaging, blood tests, a core biopsy and pre chemotherapy evaluation is the chronological order of treatment preferred to reduce the chance of contamination [38, 39], these biopsy samples are reviewed by a musculoskeletal pathologist and radiologist to design the treatment plan. The classification of each stage of diagnosis used by the Musculoskeletal Tumor Society classifies as low or high grade (I, II), location of the tumor (A/B) whether it is intra or extra- compartmental and metastases as (III) [37, 40] . The most common treatment plans employed include chemotherapy, radiation therapy, immunotherapy and surgery where each modality is accompanied with its own side effects to the host. The treatment for OS is usually a combinational approach of surgery followed by adjuvant and neo-adjuvant chemotherapy [41]. The summary of the current treatment strategies applied for osteosarcoma patients has been detailed below in Fig. 1.

### Chemotherapy treatment

Chemotherapy was not used much in the treatment of OS for a long time until trials began for adjuvant chemotherapy and the results showed improvement in the disease-free condition of patients at two years compared to the other treatment [42]. Some of the most common chemotherapeutic drugs are methotrexate with leucoverin, ifosfamide, Adriamycin/doxorubicin [43] (DOX), cisplatin [20] and etoposide [44]. Combinational therapy alongside chemotherapy has been employed to increase the effectiveness of chemotherapeutic drugs, reduces drug resistance and induces apoptosis thus shrinking the size of the tumor [45]. Thermo-chemotherapy [46](synergistic effect of heat stimulation and anti-cancer drug administration) has also been studied to see the effect of cytotoxicity, reported by [47] wherein the effect of drug cisplatin seemed to increase while temperature was also increased [48]. Many other reports with the combinational use of hyperthermia and chemotherapy at localized site have also shown good results by enhancing the anti-tumor activity or caspase-3 activation [49–51]. The first line treatment for any type of tumor has been chemotherapy in spite of its health hazards and drug resistance risks as it targets critical cancer metabolism pathways and has overall efficacy of treatment benefit for the host. Recent chemotherapy nanoparticle based therapy, called chemodynamic therapy (CDT) acts as drug delivery module followed by hydroxyl radical (OH^–^) generation using light assisted or Fenton reaction. Studies done by Cheng et.al have shown the theranostic effect of metals like Cu and Cerium conjugated to Arg-Gly-Asp (RGD) for dual fluorescence bio-imaging (NIR) and tumour ablation therapy of osteosarcoma. [15]. Thus combinational therapy has shown to be a potential route to deliver the respective drug and minimize cancer drug resistance and other challenges.

### Radiation therapy(RT)

RT is traditionally recognized as a potent method of directly killing cancer cells by DNA damage and the production of free radicals. Tumor-associated antigens (TAA) and their release are enhanced by radiation therapy (RT) and cause apoptosis in cancer cells [52]. Combinational therapy along with RT delivers the added advantages of radio therapeutic based treatment as a robust and efficient approach. One of the main limitations to radiation therapy (RT) is radioresistance (RR), which causes cancer to recur after a specific time period. Numerous advancements involving nanoparticles, combinational therapy in radiotherapeutic techniques have been implemented to maximize safety and effectiveness while reducing tumor radiation dose and also prevent radioresistance. [53].Osteosarcoma cells are presumed to be radio-resistant and progress in orthopedic surgery have made it possible to perform substantial bone resections with wider margins, followed by prosthesis implantation, hence radiotherapy (RT) is not regularly applied for osteosarcoma treatment [54]. However, when a localized disease is inoperable, radiotherapy (RT) might be utilized as a final treatment option or as an adjuvant treatment following surgery with suitable margins [55]. Hyperthermia has also been combined with radiation for the sensitivity of cells to increase the effect of irradiation as seen in one of the case reports by Tancredi et.al [50]. When there is only one or a few metastases, RT may also be a better option than surgery as radiation seems to be a better hope of treatment in OS particularly as the combinational therapy accompanied with surgical procedures can be used when it is difficult to administer chemotherapy [56] for primary tumors or patients whose conditions are not feasible or for an already irradiated site [57]. This type of radiotherapy has its own disadvantages affecting the host immune system and causing damage to normal tissues.

### Surgical resection

Tumor surgery is the complete resection of the infectious part of the cancerous bone and in severe cases can be limb salvage or amputation. Limb salvage is the excision of the tumor including the biopsy site with prior imaging of bone and CT scan followed by reconstruction [58–60]. The surgery can be limited to the particular cancellous bone and provided a safe margin to be excised by the aid of a computer based navigation in interior areas like the pelvic and sacral region [61]. Physical destruction, irregular lifestyle, improper growth of bone are some of the complications faced earlier which have been resolved to an extent by endoprosthesis, substitutes, rotation plasty and composite allograft prosthesis as a substitute [62]. In 1994, Canadell depicted a technique for consolidating interruption osteogenesis with an outer fixator with tumor resection so as to attempt to diminish development errors. They worked on 20 patients, of whom none had nearby repeats and three had pulmonary metastases. They at last decided this technique a protected and successful approach to resect tumor yet keeping up the epiphysis of long bones [63] and some simulations have also been made. Endoprosthetic replacements have proved to provide a promising result in limb salvage reconstruction in weight-bearing bones as the design is custom-made nowadays modular ones with many metal based implants [64]^,^ some still under study like titanium, cobalt-chrome alloys [41] or coated titanium prosthesis to reduce infection risk^,^ [65]. Biological replacement is the next form of reconstruction which has been in practice since 1908 including allograft, autograft or auto/allo prosthetic reconstructions. Autograft harvesting is mostly done from the fibula as it is long, tube like less load bearing [66] . Allograft prosthetic composites can be used for load bearing joints like the hip and knee but the risk of infection is quite high [67]. The recurrence rates in the case of limb salvaging seems to be quite high while the survival rates are much comparable to amputation [68]. The restoring of the lifestyle of the patients back to normal has been observed much effectively with the use of modular endoprosthetic replacements which makes it one of the most preferred treatment for malignant OS [69]

### Amputation

The next surgical treatment of OS is amputation which faces lots of controversy as it affects the patient directly in terms of survival and the restoration of daily function becomes impossible [69]. Amputation followed by osteointegration implants have shown to improve the function which has been reported by a study done by Branemark et al. who studied 51 patients, trans femoral amputees with a survival rate of 92% [67]. Rotationplasty is the rotation of the tibia to 180⁰ in order to allow the ankle to function similar to the former knee joint which is mostly carried out for sarcomas around the distal femur and proximal tibia in children and young adults when endoprosthesis replacements are not possible [70]. Reports have been made that this procedure has restored successful function status without pain and even patients returned to competitive sport eventually [69] with much improved psychological responses from patients [71].

### Photodynamic therapy(PDT) and photothermal therapy (PTT)

PDT is another non-invasive treatment strategy based on photosensitizer (light activated agent) injection followed by light irradiation with a controlled wavelength between 600 and 800 nm near the infra-red region. This modality is two stage treatment that damages or kills tumor cells through the use of photosensitizers, light energy activating reactive oxygen species (ROS) such as •OH, singlet oxygen (^1^O_2_ ,excited state of molecular oxygen) and superoxide anion (O_2_^●–^) [72] to induce cell death. Reactive oxygen species (ROS) are produced by cells naturally to carry out essential functions such as cell interaction and defense mechanism against pathogens; within a normal physiological system ROS have beneficial effects, including the regulation of gene expression, cell differentiation, and stem cell maintenance. When reactive oxygen species (ROS) levels exceed the physiological range, they can become harmful to cellular components, including nucleic acids, lipids, and proteins, causing cellular disruption, necrosis and apoptosis [11]. PDT and PTT (Photo thermal therapy) deliver a localized treatment where the cell destruction is limited to the photosensitizer’s located region. Therefore, PDT shows better selectivity and fewer side effects than conventional chemotherapy causing less harm to the healthy tissues. [73]. The reaching of deep tissues is a challenge with the effect of the appropriate drug efficacy. They are different from photothermal therapy as the photosensitizers do not cause hike in temperature and remain non-toxic to cells in the dark [74–76] however it is essential to mention that the presence of oxygen levels influence the delivery of light [77] and efficiency of apoptosis [21]. The combinational therapy of PDT along with radiotherapy, immunotherapy and chemotherapy has been used to reach deep tissues. Designing vehicles carrying photosensitizers conjugated to anticancer drugs has been a key area of research recently for targeting hypoxia based release. X-Ray induced Photodynamic therapy (X-PDT) using nanoscintillators and photosensitizers possess the capability to absorb X-rays to reach deep seated tumors for excitation followed by generation of ROS [11].A clinical trial using Acridine Orange (AO-PDT) was conducted on 10 patients with primary musculoskeletal sarcomas (OS-two patients), and the results indicated that all patients' limb function returned almost to pre-surgical level(except 1) and no patient experienced adverse complications [78]. Recent study using Fe metal-organic framework conjugated to erastin (ferroptosis inducer) for PTT showed increased ROS and tumour suppression in OS bearing mice using allograft models [12]. These findings suggest that PDT and PTT have a low toxic effect and could be a novel option for conjugated therapy. There are many studies going on to implement PDT as an efficient combinational therapy treatment plan**.**

### Ultrasound based adjuvant therapy

The application of ultrasound for cancer therapy for diagnosis and treatment has gained focus for different tumors including liver, glioblastoma, bone, breast and prostate cancer [79]. It is also commonly known that mechanical stimulation, like ultrasounds, in the bone stroma are crucial to preserve bone development and homeostasis and are essential for the development of tissues, including lung, cartilage, and bone [80]. Lower-intensity ultrasounds can also contribute to the development of a moderate temperature, which improves blood circulation and stimulates vascular permeability. These properties are beneficial for bone regeneration and the treatment of tumors. In this way, higher temperatures and oxygen levels enhance the efficiency of chemotherapy [81]. Low intensity Pulsed ultrasound (LIPU 20 to 200 KHz) and High Intensity Focused Ultrasound (HIFU 1–20 MHz) are among the two types accompanied by Sono dynamic Therapy (SDT) to focus targeted tumor site for activation followed by chemotherapy [82]. Recently the combination of MRI-based High Intensity Focused Ultrasound (HIFU) has been recommended by FDA for pain management in bone tumour metastatic patients [83]. Besides the studies conducted on 2-Dimensional cultures to observe the effect, research is moving towards 3-Dimensional analysis of physical stimuli for diagnostic and therapeutic purposes. Iron doped ZnO has been used as a targeting agent using peptide conjugate of erythropoietin-producing hepatocellular receptor – 2 (EphA2) for rapid internalization and sonodynamic approach assisted anti-cancer effect using a 3D spheroid model [84]. These methods of adjuvant therapy serves as a less invasive and more tissue focussed target implicative therapy.

### Combinational therapy: upcoming intervention

The combinational therapy involves the use of two or more therapies listed above in moderation with respect to severity of the condition, diagnosis stage, age, underlying chronic diseases of the patient implying the treatment plan by the oncologists. Different combinations are being evaluated and some are still under clinical trials for improving the efficacy of treatment. Many clinical trials have shown that radiation therapy along with chemotherapy or hyperthermia have shown effective results in treatment and management of osteosarcoma [53]. Hyperthermia (HT) is the induced increment of temperature in target cells inside a scope of 39–45 ⁰C. It is believed that ancient medicine involved the treatment using heat for various diseases. Eventually around the 19th century it gained interest and was explored on a clinical level and was thus observed that cells were more sensitive to hyperthermia [85].

**Fig. 2:**
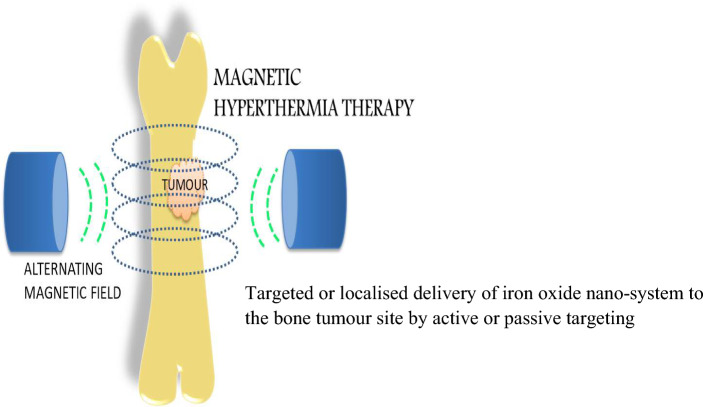
Illustration of mechanism of hyperthermia using external magnetic stimulation (Inspired from [33, 49, 88])

At tissue level, this expansion changes the vascular porousness, upgrades blood stream and could prompt oxygenation of the tumor, making the malignant growth cells increasingly vulnerable to other treatment modalities thereby increasing the effect of the drug or radiation [42]. Traditional HT produces a temperature slope with a most extreme on the body's surface that diminishes while moving ceaselessly from the source; in this manner, most of vitality is scattered in the solid tissues arranged along the way outside radiation with no separation between the focus on tissue and the encompassing ordinary tissues arranged along the way of outside radiation with no separation in between [86]. Thus the increase in temperature results in annihilation of the tumour tissues also resulting in mild damage to the surrounding healthy tissues. It has shown good results while the normal tissues have to be protected from heat burns. Hyperthermia has been used for combinational therapy with chemotherapy, radiation and Photo dynamic therapy as stated above. In thermo-ablation the cells are exposed to very high temperatures >56 ºC forcing the cells to undergo cell coagulation and necrosis. The range between 40 and 44 ºC is termed as moderate hyperthermia and below 41 ºC called diathermia which is used for treatments like physiotherapy [87]. Many *in-vitro* and *in-vivo* studies have been done and reported where coated MNPs have been used in various ways to study the internalization and thermo-responsive changes where the iron oxide nanosystems are directed towards the tumour site followed by application of an external magnetic field as illustrated in Fig. 2 [33, 88]. Phosphate-Starch coated MNPs were combined with positively charged cisplatin for drug release and cell inhibition using hyperthermia [86, 87, 89]. The application of hyperthermia towards treatment of osteosarcoma has been studied using nano-engineered PCL polymeric scaffolds loaded with metal nanoparticles for photo-thermal ablation of OS and also promotion of bone repair [90]. Zhang et.al reported the use of RGD modified silica based Fin56 loaded iron oxide nanoparticle for laser assisted localized hyperthermia and ferroptotic death of osteosarcoma cells in vitro and in vivo (Female athymic BALB/c nude mice). The first clinical trial was done in glioblastoma and prostrate carcinoma by the research group of Jordan et.al [91] with a frequency and amplitude adjustable magnetic hyperthermia device (Field-0–15 kA/m, frequency-100 kHz). Later along with the company Magforce, they fabricated the prototype of the magnetic induction based hyperthermia machine with a cylindrical area of diameter 20 cm and height 300 mm and magnetic field of 12 to 18 kA/m, which has been under trials for application in clinical treatment [92]. The translation of combinational therapies with PDT, PTT, chemotherapy, radiation therapy for osteosarcoma is under rigorous research as an efficient tumour management modality. Presenting all the various present clinical therapies being involved towards the aim of improving treatment and lifestyle for the affected patients, below are some of the reasons why magnetic nanoparticles are being investigated towards this direction.

## Interospection of magnetic nanoparticles as a therapeutic strategy for cancer nanomedicine

### Background

**Fig. 3:**
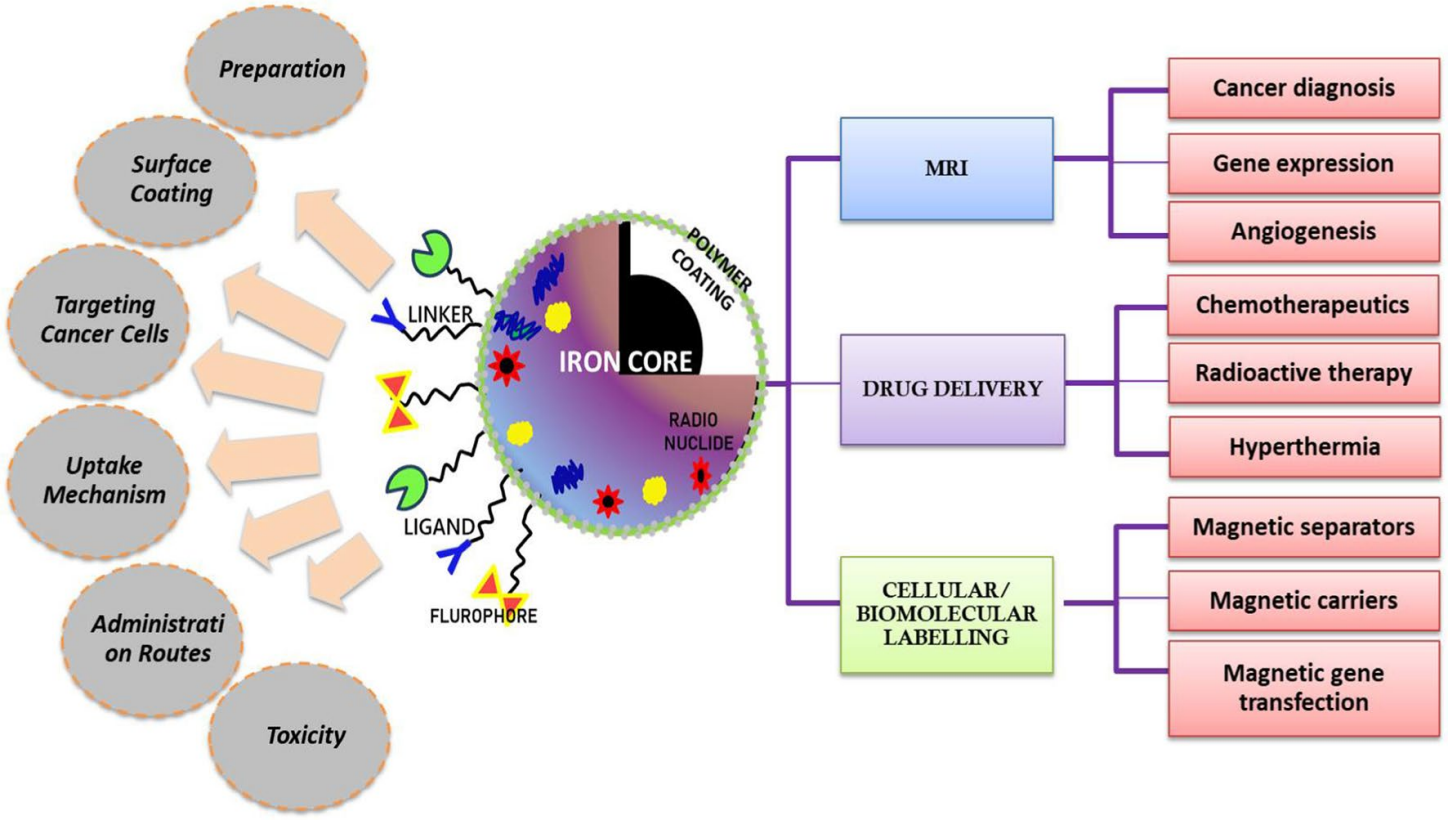
Modification of magnetic nanoparticles and their application in cancer therapy

Nanotechnology in cancer medicine has paved a new way to address the challenges faced in the present medical and surgical practices for treatment. The detailed study and prolonged research conducted using nanoparticles aims to intensify the attenuation of cancer cells with minimal drug exposure, reduced recurrence and to attack the tumor site through less invasive and potential drug delivery methods using both active and passive targeting methods where many metallic, organic and inorganic compounds are being extensively researched to achieve the same [61].The clinical insights, experimental results, and acquired data obtained over the last few decades focused on tumor biology, further investigated at the molecular level, has led to an improved, precise, and more efficient therapeutic strategy referred to as nanomedicine, which aims to reduce the conventional problems encountered thus far. The goal of nanomedicine is to treat diverse conditions by applying diverse qualities of nano-systems and tuning them to the specific requirements of the issue at hand. Iron oxide nanoparticles are an essential metal to be investigated in medical therapies and diagnostics due to their inherent magnetic characteristics(Fig. 3) The iron oxide nanoparticles exhibit a phenomenal behavior termed as ‘super-para magnetism’, which behaves similarly to paramagnetic materials but with higher magnitude because single-domain nanoparticles behave as single entities with larger magnetic moments; owing to this the sub-domain particles align themselves to the corresponding direction of applied magnetic field. The crucial reason for their wide focus in biomedical application is after application, the magnetic field is removed they recover back to their original random orientation domains without any residual magnetic field [93]. The aforementioned phenomenon occurs due to the change in the spins of the particle which dissipates the energy in the form of heat. The heat produced in the particles when placed in an Alternating Magnetic Field (AMF) is due to the Brownian effect or Neel effect [94]. This increase in temperature is used to kills the cancer cells at a particular site as the cells inside tumorous site are more sensitive to increased temperature conditions than the normal cells.

The main issues to be addressed whilst using the magnetic nanoparticles within the body are agglomeration, biocompatibility, toxicity, drug resistance, normal cell behavior and reducing side effects along with effective treatment to block the growth and proliferation of carcinogenic cells [95]. The use of these particles in the bare form could lead to agglomeration, produce cytotoxicity to healthy cells and might affect the pharmacokinetics of the delivery system within the body. Due to this various kinds of surface modification and functionalization has been attempted on magnetic nanoparticles to improve its colloidal stability, increase circulation time, enhanced targeting efficiency and controlled drug release mechanism.

### Various synthesis methods of iron oxide nanoparticles

Chemically different phases of iron oxide like magnetite, haematite, maghemite have been studied as the morphological and physico-chemical properties are strongly based on the synthesis procedure, where Magnetite (Fe_3_O_4_) and Maghemite (Fe_2_O_3)_ have been identified to have the most favorable properties and biocompatible conditions for further translational study [96]. There are various physical, biological and chemical methods of both top-down and bottom-up approach synthesis of iron oxide nanoparticles. The different chemical methods of synthesis of magnetic nanoparticles employed so far includes sol–gel, co-precipitation, sono-chemical, micro-emulsion, thermal decomposition and more.

**Fig. 4:**
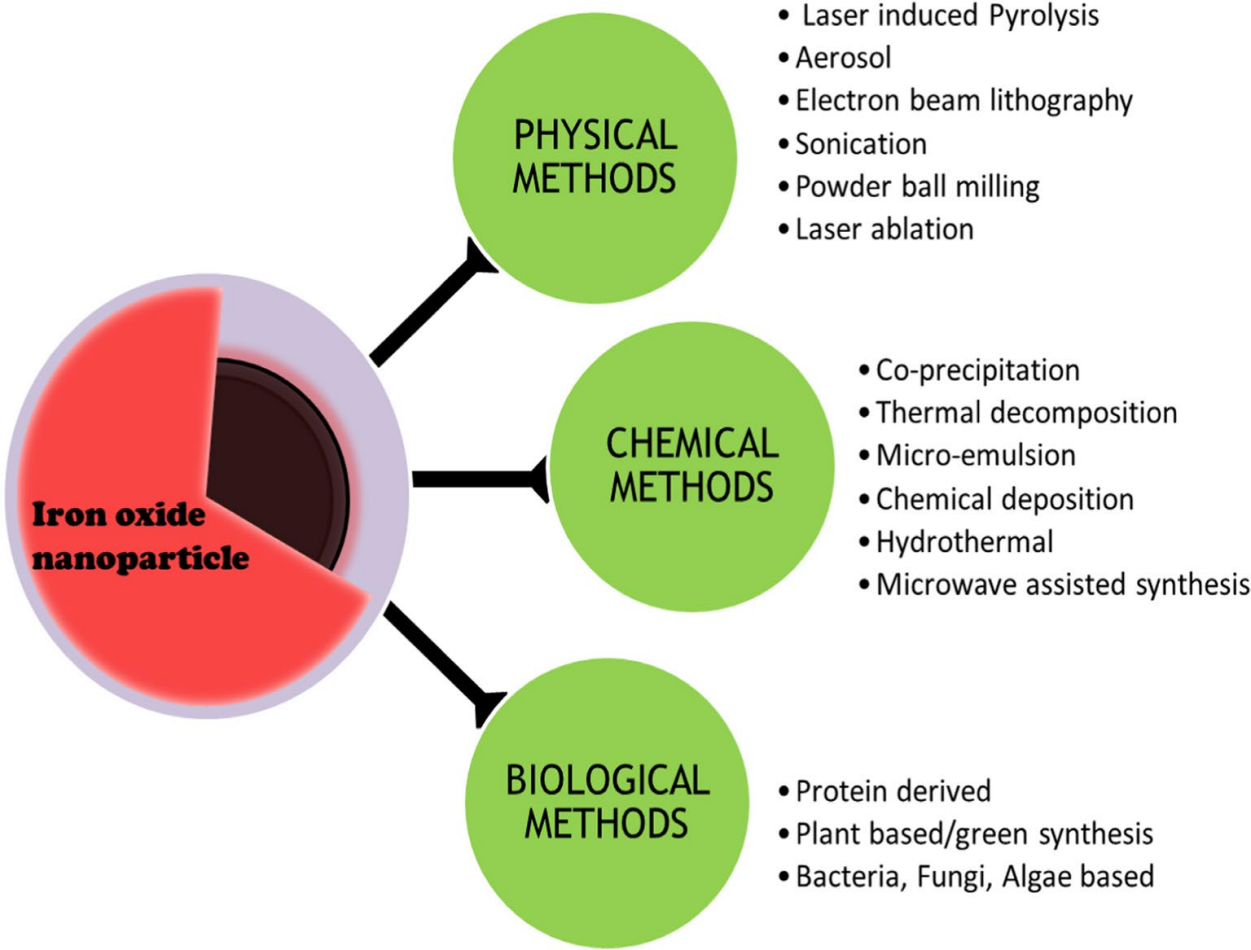
Synthesis of magnetic nanoparticles using various physical, chemical and biological methods

Some of the conventional methods used so far have been represented in the Fig. 4 and explained shortly below.

*Co-precipitation* is a simple and practical way; most common methods of synthesis followed for bulk production and controlled size of iron oxide nanoparticles. The chemical process is based on the addition of NaOH solution to a salt solution to reduce metallic components, either at ambient temperature or at increased temperature. It has been demonstrated that the size, shape, and composition of magnetic nanoparticles rely on the salt type utilized, the reaction temperature, the pH level, and the ionic strength of the media. There are many reports on the synthesis of magnetite using this method with novel approaches in view of biomedical application [18, 19]. The quality of the magnetic nanoparticles can be replicated though once the synthesis conditions are fixed [97].

*Thermal decomposition* is one of the techniques which require high temperature condition to be carried out [19]. With surfactants like oleic acid and hexadecyl amine there have been attempts for controlled size and morphology with respect to reaction time conducted [98]. The particles have a uniform size distribution and so are used in applications like MRI.

*Sol-gel technique* is a wet route for preparation, very suitable for synthesis of uniform size-controlled particles. Initially hydrolysis and condensation reaction of metal alkoxides forming a ‘sol’ and later inorganic polymerization called ‘gel’ finally removed by heating at a high temperature to obtain fine crystalline powders [76, 99]. Ferric Nitrate and ethylene glycol were used to prepare non-toxic magnetite by annealing at 200°, 300° and 400 ⁰C [100], which clearly indicated that increase in temperature primly affected the size. The purity, amorphous nature and determined structure can be tuned by this method effectively.

In the *sono-chemical method* it is possible to generate small uniform sizes based on the impulse from the acoustic irradiation which helps in particle formation through the ultrasound probe and has been reported [101], where 10 nm sized particles were formed. The sonication of iron carbonyl in decalin and annealing at 350 ⁰C under Nitrogen gas atmosphere resulted in the formation of an amorphous form of iron oxide with size 50 nm [102]. The high temperature generated from the ultrasound radiation causes decomposition of the salts [103] used resulting in improvised hydrophilicity, high electron storage capability and uniformly disperse nature, [104].

*Green synthesis* has been used for synthesis of MNPs using natural extracts as substitutes to chemical methods aiming to be bio-compatible and economical displaying several advantages compared to conventional methods [105]. Some of the extracts used from plants like *Magnifera indica* (mango), dates, *Garcinia mangostana,* eucalyptus, *K. alvarezii* [106], *Hibiscus* [107] dried fruit extract of (Fig)*Ficus carica* and sugarcane juice. The composition of the extract plays the role of reducing agents and also capping and stabilizing agents during the chemical reaction. Monodisperse particles with good stability and less toxicity are produced which provides a promising future for use in biomedical applications.

*Hydrothermal method*: This approach yields metal precursors in an aqueous media without the usage of surfactants. They undergo treatment to high reaction temperatures and pressures in an autoclave. When a reducing agent, such as ammonium hydroxide, is introduced drop wise to an aqueous solution containing Ferric and ferrous chloride, the solution is heated to the required temperature using argon or any other inert gas and vigorously stirred [100]. The reaction mixture is placed in an autoclave for a certain amount of time for drying before which it is rinsed numerous times with deionized water to eliminate any excess ions or salts. Surfactants are used to avoid or lessen aggregation when synthesizing such NPs using the hydrothermal approach. The synthesized particles have uniform shape and precise size distribution ranging below 100 nm for higher coercivity and magnetic saturation based application.

The other common methods include electrochemical synthesis which grow or deposit iron oxides on other materials on other polymeric structures [108], polyol method which involves the use of diethylene glycol, trietheylene glycol, and tetraethyelene glycol under different reflux temperatures [109], micro-emulsion (oil phase and water phase emulsified in the presence of a surfactant for mono disperse and coated iron oxide nanoparticles) [110] wherein the influence of the synthesis parameters such as temperature, precursors, pressure, capping agents, reaction time [111] can be modified according to the targeted application while each have their own advantages and disadvantages [112].

### Physico-chemical properties and surface functionalization of iron oxide nanoparticles for cancer application

The important features to be noted for the transition of magnetic nanoparticles to clinical application is its size, shape, crystallinity, and polydispersity in a colloidal solution as generally the particles tend to form aggregates and settle down [113]. The effect of size and shape, surface charge, precursors used for synthesis, concentration plays an important role in the reactivity, stability, zeta potential, aggregation property, anti-bacterial activity, absorption and cellular uptake [114]. Iron oxide nanoparticles have also been doped using transition metals, rare-earth elements and other elements like cobalt, nickel to improve its opto-electric, chemical and biological properties [115]. Degradation is also one among the prime conditions that requires attention as these iron oxide nanoparticles might produce free radicals in cells, causing damage and cell death [116]. Tailoring of the surface coatings for the iron oxide nanoparticles has been a key interplay to unlock the contribution and potential of magnetic nanoparticles towards biomedicine. These modified surfaces provide a wide and new dimension for conjugation of various drugs, targeted controlled drug release mechanism, enhanced circulation time within the body and many advantageous strategies to overcome the challenges faced in precision medicine [112]. Additionally, another drawback to be rectified of the magnetic nanoparticles is their high oxidation chances, bio toxicity towards normal cells and their non-specific binding to the cell-components and biological systems along with the colloidal instability causing accumulation in inappropriate tissues [117].

Their extremely small size can cross the blood brain barriers and get into the various body systems causing malfunction or damage neural functions also causing mutations if they go beyond the nuclear membrane. The agglomeration of bare particles can lead to an obstruction in the blood vessels [118]. For the transition of magnetic nanoparticles into the clinical setup rectification of all these major issues directs research to the need for a proper surface modification using an appropriate material to improve its physicochemical properties, reduced oxidation and toxicity, enhanced biocompatibility, colloidal stability and delayed degradation within the body [111].

The common structure is the core–shell model of magnetic nanoparticles where the core magnetic component is surrounded by a polymeric coating. The surface can be further modified using other biomolecules, drugs, functional ligands, markers, to be attached to the functional groups of the polymer used. Among the feasible coating materials, silica has been most widely and commonly used as it has exhibited better biocompatibility, non-toxicity and facilitates surface modification through covalent bonds [119]. Carbon-based materials have been used for coating to improve the dispersity and hydrophilicity. Nano composites like Fe_3_O_4_/CNT prepared have been used for the dual-drug delivery of drugs like epirubicin hydrochloride and paclitaxel for cancer treatment [120]. Other coating materials utilized include organic coatings like dextran [19], starch[74], gelatin, PEG(poly ethylene glycol), silk fibroin, poly(D,L-lactide). The application of iron oxide nanoparticles as contrast agents for MRI applications have been explored as they have the capability to increase the proton relaxation time in tissues [95].

### Drug targeting strategies and uptake mechanism in cancer cells

**Fig. 5:**
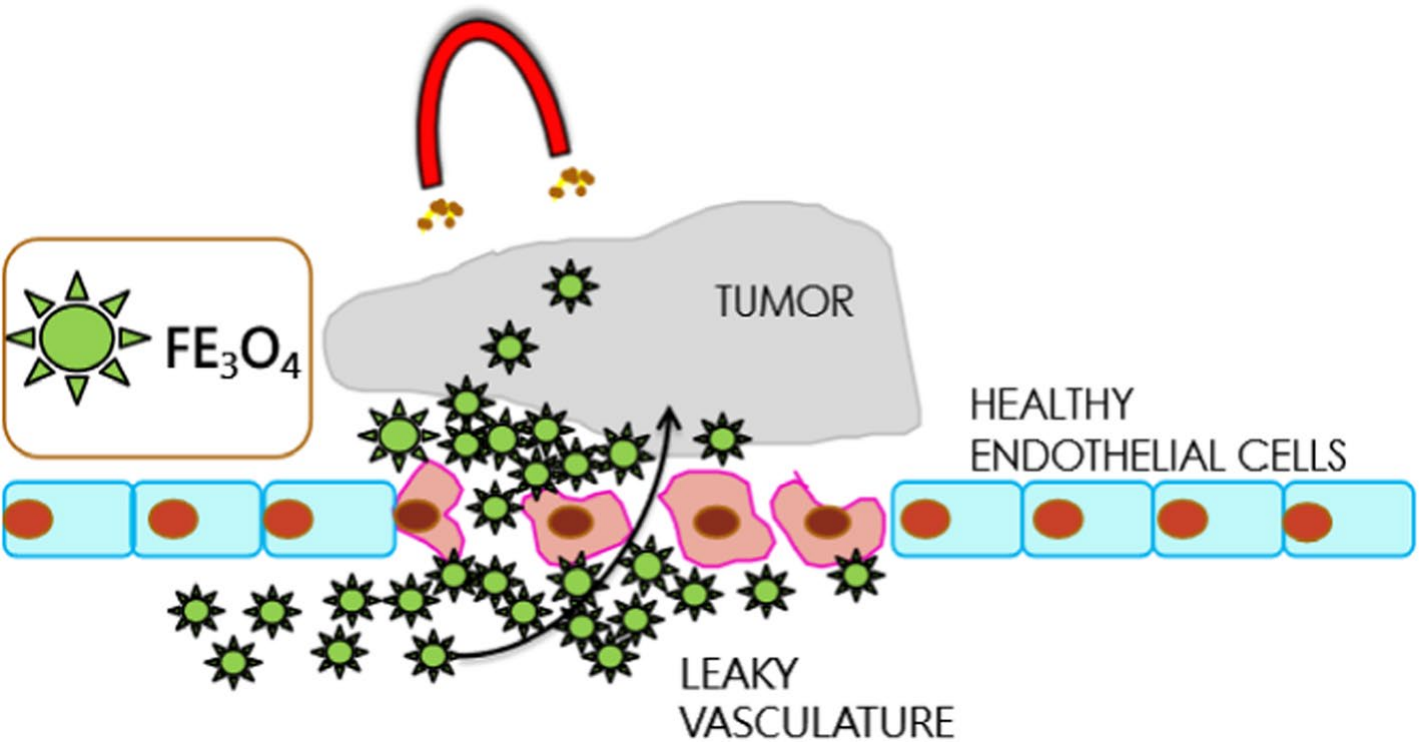
Schematic representation of magnetic field based uptake in tumour cells via EPR effect for passive targeting [121]

The delivery of these particles also plays an important role as direct intra-tumoral injection is quite complicated, which was first reported using 100 kHz machine on prostate cancer patient and also later done in glioma patients [23] served the purpose of reducing the toxicity and attaining maximum concentration yet did not effectively destroy the entire tumor cells due to the inhomogeneous distribution. Stimuli-based drug delivery is also being developed for precise and selective drug delivery that is triggered by factors like pH, enzyme, light, magnetic field, redox etc., that provides the required efficacy in treatment [44, 48]. Since the pH of tumor is different from the normal healthy cell, the pH sensitive carriers rapidly release the drugs when there is a change in the surrounding environment [121]. Due to this the targeted nanoscale drug delivery plays a very promising role in the prognosis of metastatic tumors. The MNPs interact with the cells by 1) passive diffusion 2) Receptor mediated endocytosis 3) Clatherin mediated endocytosis and 4) caveoline mediated endocytosis [77]. Once the particles enter the cells, they are degraded by lysosomes to form ions which generates reactive oxygen species (ROS) by changing mitochondrial functions and inducing the cell signaling pathways [116].There are several types of nanocarriers used in the targeting of the multifunctional nanoparticles’ delivery of drugs to the specific site using the active and passive ways. The active targeting strategies like ligand-based delivery can interact with the cancer cells which result in uptake of particles by endocytosis leading to a greater therapeutic effect. In this pathway of targeting the site specific particles reach by molecular recognition [122] whereas in passive targeting the leaky tumor vasculature plays the role where the particles accumulate due to enhanced permeability and retention effect (EPR) [123] shown in Fig. 5 as magnetic field based uptake inspired from Tian et.al [124]. Since apoptosis is deactivated in cancerous cells they rapidly keep proliferating and dividing at an uncontrolled rate by absorbing the nutrition from the neighboring blood vessels thus leading to neo-angiogenesis causing the particles to easily penetrate through the crevices to reach the tumor site [125]. The targeting of the particular cells can be facilitated using binders to specific biomarkers on the surface of the cancer cells. The ligands used for targeting can be proteins (antibodies), nucleic acids, or peptides, vitamins, carbohydrates which bind directly to the site and also enhance uptake of the particles by endocytosis, pinocytosis or micropinocytosis [126]. Owing that a tumor's pH differs from that of a healthy cell, when the environment around it changes, the pH-sensitive carriers quickly release the loaded drug.

### Toxicity aspect and translational strategy

Toxicity aspect of the iron oxide nanoparticles is one of the most fundamental and integral developmental step towards translation of the nanoparticles to clinical approval. The toxicity caused by the magnetic nanoparticles could have a direct impact on the plasma membrane and proteins by activating the proteins which is responsible for apoptosis and activation of stress. Considering the potential effects and applications topical research on toxicological perspective of the synergistic magnetic nanosystems in cell based in-vitro and animal based have been studied under many reports. The identified routes of delivery play a major role in toxic response such as injection-intra muscular, intra pulmonary inhalation and intestinal tracts. In a study, sequential exposure to iron oxide dust in rats were observed to have no change in their body weight, no systemic toxicity or any drastic changes [127] while exposure of iron oxide nanoparticles to murine macrophage cells showed higher apoptotic rate caused by intracellular ROS generated by the magnetic nanosystems [128]. The toxicity of iron oxide was evaluated on eggs and zebrafish by [129] where teratogenicity of Fe_2_O_3_1600 mg/L revealed no distortion, 100% of the embryo hatched after 96 h. The toxicity of SPION coated with cross-linked aminated dextran (CLIO-NH2) was examined for effect of the exposure time (1, 16, 24, and 48 h) and at various dosages (20, 50, 100, 140, and 200 mg/kg) on the zebrafish brain. The results pointed to immediate brain damage brought on by acetylcholinesterase inactivation and apoptosis activation [130]. Dextran has been clinically approved though with lower concentration of dosage [131]. Bovine Serum Albumin (BSA) and citrate coated iron oxide nanoparticles with a concentration of 100 µg/ml has shown a slightly toxic effect on Caenorhabditis elegans [132]. Au-Fe_3_O_4_ composite magnetic nanoparticles reported that at concentration of 100 mg/ml most of the particles were ingested in mice with no inherent toxic effects to mice bone-marrow cells and beagle dogs and were considered to be a safe use in a biological system [133].Cobalt ferrite nanoparticles coated with chitosan has been studied for acute toxicity and applicability as an MRI contrast dye in albino Wistar rats. The time dependent (1-28 days) and concentration based evaluation shows contrast enhancement up to 20 mg/kg (corresponds to 3.24 mg/kg human dose) with no significant toxicity effect [134]. Additionally magnetic albumin nanospheres with coated γ-Fe_2_O_3_ demonstrated good biocompatibility at a dose of 5 mg/ml in mice model [135]. The employment of natural coating and functionalization on iron oxide nanosystems could be a pioneer to show better effects to overcome toxicological effects in biological systems.

## Bio-polymer conjugated magnetic nanoparticles for osteosarcoma treatment and diagnosis

Magnetic nanosystems modified using natural sources of origin are among the most favorable for precision medicine in the current medical scenario as they are based on natural genesis such as sugars, proteins, polysaccharides, peptides, nucleic acids derived from plants, animals or other biological resources, displaying a much improvised scope to be translated within the body system [22] with a greater potential significance. Nanoparticles have poor penetration into osteosarcoma tissues due to its strong extracellular matrix, while it also minimizes increased permeability and retention (EPR) effect. Therefore specifically improving the permeability of these tissues is crucial for an efficient targeting and drug delivery [136]. The development of materials based on naturally occurring polymers has gained impetus recently because of their unique features including their low cost, ease of preparation, and remarkable stability in physiological fluid; they also exhibit improved biocompatibility, increased circulation time, binding, and proliferate with great efficiency which make them specifically beneficial for a range of biomedical applications [137]. Certain biopolymers exhibit advantages such as being elements of the extracellular matrix found in nature (collagen),cellulose [138], gelatin [139], hyaluronic acid present in the synovial fluid [140] branched polysaccharide polymers like alginates [141], starch [142], globular protein like albumin [143], dextran [144] or possessing antibacterial and repellent characteristics (chitosan) [145]. In addition to their biological system natural biopolymers have functional groups such as hydroxyl, amino, or carboxyl in their structure; they are as versatile and have robust chemical properties as synthetic polymers. In particular, these reactive groups allow modifications that improve the long-term stability of biopolymers in biological conditions [146]. Because natural polymer-based products are non-toxic and biodegradable, their application is also advantageous for the environment where they can be applied in agriculture or to eliminate metallic ions from water-based environments [147].

Consequently these natural components designed as liposomes, drug delivery agents, polymeric nanoparticles, hydrogels, dendrimers, nanomicelles, exosome-based, nanocapsules, imaging agents, photodynamic and hyperthermic activity are explored conjugating different cancer moieties encapsulated over the iron oxide nanoparticles so as to dodge the EPR retention mechanism by the body to flush out a foreign body from the system (Table 1). The incorporation of natural polymer coating on iron oxide nanoparticles results in a sensitive magnetic material with a less toxic and immunologically stable coating responsive to external magnetic stimuli for remote control drug release feasibility [131]. Targeted drug delivery agents like CD80 and VEGF growth hormone conjugated with magnetite was investigated on osteosarcoma cell line ATCCM CRL-2836 cells [148]. There has been use of Fe-hydroxyapatite (HAP) and functionalized with microRNAs has been studied to improved cell viability and cell proliferation in murine pre-osteoblasts MC3T3-E1 and pre osteoclasts 4B12 [149]. Dextran and Polyethylenimine (PEI) coated iron oxide nanoparticles were studied by Gong et.al for the delivery of miR-302b targeted towards YOD1deubiquitinase (YOD1) and to inhibit osteosarcoma with cytotoxicity as a major concern. [150]. However Lin et.al created a PEI-chitosan and iron oxide crosslinked polymer with improved drug loading efficiency and DNA delivery in breast cancer cells. This section covers the prominent natural composition with iron oxide nanoparticles in various nanoplatforms aiming to address the research gap and glitch faced for osteosarcoma treatment.

**Table 1:** Different natural polymers and biological moieties used for coating magnetic nanoparticles targeting osteosarcoma and their strategy

Biomaterial (Natural source)	Modification	Significance and observed effects	References
Gelatin	Self—assembled nanocapsule	Burst release of vitamin B12 under magnetic induction	[154]
	Targeted drug delivery	Genipin crosslinked magnetic carriers of doxorubicin for magnetic field and pH based release	[155]
	3D porous scaffolds	Gentamicine release from magnetic gelatin based porous scaffolds	[156]
Silk fibroin	Magnetic e-gel scaffolds	Silk fibroin e-gels with human serum albumin/iron oxide loaded with fibroblast growth factor studied for osteoblast differentiation	[157]
	Freeze dried 3d magnetic scaffolds	Silk fibroin and chitosan based magnetic scaffolds on Mg-63 cells under static magnetic field	[158]
Chitosan	pH sensitive nanocomposites	Chitosan-folic acid conjugated with doxorubicin loaded magnetic ferrofluid studied on MG-63 and lung cancer A549cells	[159]
	3d printed aerogel scaffold	Silk fibroin with MXene to enable photothermal ablation and heat based drug release	[155]
	Hydrogel	Silk fibroin hydrogel loaded with Fe_3_O_4_ and polyacrylic acid for osteogenic differentiation of MSCs	[160]
Dextran	Magnetic gene carrier	Polyethylenimine, dextran and iron oxide for miR-302b transfection in OS cells and OS-bearing nude mice	[161]
Hyaluronic acid	Redox sensitive and targeted liposomes	Hyaluronic acid, polyethyelene glycol2000-chol loaded with doxorubicin showed higher internalization in MG-63 over liver cells LO2	[162]
Starch	Antitumour cargo vehicle	Assam bora rice starch loaded with doxorubicin studied in MCF-7	[163]
	Drug loaded nanoparticles	Amoxicillin loaded starch coated magnetic nanoparticles for osteomyelitis	[164]
Alginate	Anticancer drug delivery vehicle	Sodium alginate-PVA-bovine serum albumin coated Fe_3_O_4_ loaded with doxorubicin	[165]
	3D hydrogel	Sodium alginate, PVA, HA loaded with magnetic graphene oxide for bone mesenchymal stem cell differentiaition	[166]
Albumin	Nanospheres	Albumin coated γ-Fe2O3 demonstrated good biocompatibility at a dose of 5 mg/ml in mice model	[135]
Biotin functionalised Exosomes	Nanocarrier	Magnetic guided photothermal therapy with miR-21 targeting agent and drug doxorubicin *in-vitro* and in vivo models	[167]

There have also been bioceramic scaffolds with β-TCP-iron oxide and graphene oxide nanocomposite layers which showed more than 75% cell death in osteosarcoma cells and significantly simulated rabbit bone marrow stromal cells(rBMSCs) to proliferate [151]. Mesoporous silica nanoparticles with a magnetic core loaded with siRNA capped with tannic acid have been explored as genetic delivery vectors for osteosarcoma [152]. BMP, VEGF and many other moieties included to improve the capacity of 3D printed magnetic scaffolds that are both photo- and magneto-responsive may be used in osteosarcoma post-surgical excision for bone regeneration. Multifunctional "all-in-one" 3D-printed composite scaffolds, with Calcium, were used to achieve self-sufficient synergistic activity for osteosarcoma inhibition. The scaffolds can generate H_2_O_2_ by the loaded Calcium oxide, which participated in the Fenton-like reaction to produce high-level ROS with the existence of iron oxide. This strategy was used to enhance the efficacy of adjuvant radiotherapy based on the intrinsic strong interaction with X-ray materials [153]

Hydrogels are also a good source of drug delivery and porous structure but lack the mechanical strength which is a limitation in bone tumor applications for not which studies is going on using natural hydrogels [168]. Studies have shown that alginate naturally inhibits the growth of OS cells by binding to cell membrane receptors and activating cancer suppressors, which strengthen the anti-inflammatory and antioxidant properties of OS patients and also enables gradual pH based drug release through a "swelling-dissolution-erosion" mechanism. [123]. Since targeting osteosarcoma using magnetic-field responsive stimuli is being researched from all angles currently; there are limited number of resources till date. The bioactive molecule coated iron oxide nanoparticles could be a potential smart targeting strategy in the future.

## In vivo and pre-clinical trials

Contribution of the iron oxide nanoparticles have been explored in many cell types including osteosarcoma cells and animal models. Iron oxide nanoparticles undergo surface modification using biomaterials with controlled size, shape and dispersiblity to improve the colloidal stability, uptake, retention and clearance. The response of each environment with respect to its biocompatibility, toxicity, body clearance and biodistribution differs prominently. It has been reported that some short iron oxide nanoparticles aggregate in the liver while longer morphological particles get accumulated in the spleen which could have long-term toxicity effects or other side effects due to increased Fe content in the body [169]. Chitosan-PLGA(poly D,L-lactide-c-glycolide)-γFe_3_O_4_ based particles were studied in BALB/c mice which showed chitosan coating aided rapid uptake in the liver and spleen within 30 min according to the MRI results [170]. Ferumoxytol based MRI was designed to see T_2_* relaxation times to identify osteonecrosis [171] in transplanted porcine brain without affecting its recovery which is one of the most investigated also only FDA approved iron oxide nanoparticle [172]. Magneto-thermal therapy has been applied in PMMA based magnetic bone cements was carried out in New Zealand rabbits without any observed side effects and strong mechanical support for the bone [173].The toxic consequences of the nanoparticles affecting the embryo development, mitochondrial function and the ability to cross the placental barrier was studies using chitosan coated cobalt iron oxide nanoparticles in pregnant albino Wistar rat model. The placental crossing of these nanoparticles did not exhibit significant pathological changes to the foetus or maternal organs while the biochemical data showed an acute nephrotoxic effect requiring a more time elaborated animal model study [174].

RANKL signaling has been one of the important factors in the proliferation and multiplication of osteosarcoma [175] leading to the investigation of Denosumab a complete human monoclonal antibody against RANKL which has showed promising results for giant cell tumor of bone(GCTB). A translational phase II clinical trial was also done by COG involving the use of anti-GD2(disialologanglioside) antibody against neuroblastoma also targeting osteosarcoma with high level expression of GD2 [176]. Parental Fe therapy has been approved by the FDA (NanoTherm®) product for patients with less hemoglobin levels, anemia and end stage renal disease patients and metastatic cancer conditions. More recently it is also being used in MRI applications. Based on some of the clinical and pre-clinical trials, the mechanism of iron in anti-tumor immune stimulation is a possibility as reported by Korangath et.al showing a consistent decline T-cells after exposure to starch coated iron oxide nanoparticles followed by infiltration into tumors associated to growth inhibition [177]. ThermoDox® is under phase III clinical trials for hepatocellular carcinoma for radiotherapy assisted treatment. A phase I clinical trial of iron oxide nanoparticles for neo-adjuvant therapy of osteosarcoma patients without limb retention treatment is under process since 2020 [178]. The biological interaction of the nanoparticles while transitioning from a cell environment to an *in-vivo* system is influenced by physical, biological, pharmacokinetic parameters of the nanosystems and the host response. The clinical trials of iron oxide nanoparticles for osteosarcoma are yet to be explored for magnetic stimuli based treatment. Thus more experimental focus on in-vivo studies is required to get a more vivid and clear picture towards the response of the host body in order to translate the use of iron oxide nanoparticles in precision cancer treatment.

## Future prospects and conclusion

Conventional surgery combined with neoadjuvant chemotherapy remains the most common treatment for osteosarcoma, despite various disadvantages, including an increased risk of infection from immunosuppression, side effects, and the possibility of recurrence. Nanoparticle-based therapeutics has the potential to significantly improve conventional approaches. Focusing on the use of magnetic nanotechnology for osteosarcoma, there is an urgent need to address numerous challenges in order to improve patient survival after surgical resection, deliver specific anti-cancer drugs to metastatic tumor sites, manage host response and side effects, as well as combat chronic effects such as drug resistance and aggressive metastatic recurrence. Magnetic nanoparticles have been extensively investigated for their potential use in biomedicine and cancer therapy. Their inherent and advantageous properties, due to superparamagnetic iron oxide, have attracted major attention to imaging and therapeutic applications. The spatial properties of ligands, as well as their ease of conjugation, could influence their targeting ability. A number of studies have been conducted to assess the uptake mechanism, circulation time, retention and excretion from the body, as well as the toxicity rate, the fate of magnetic nanoparticles over time, magnetic field-based tumor destruction, thermal ablation, targeting effect, drug delivery efficiency, tissue imaging, and a variety of other cancer-related applications.

The primary issue to be addressed, along with the implications of iron oxide nanosystems, is the practical translation of research from the scientific to the clinical and commercial phases. One of the reasons for the delayed translation to the clinical forum is a lack of understanding of the interaction of the biological system with nanosystems, host response, physical parameter-based pharmacokinetic alterations, metabolic pathways, and excretion. Although surface coating and functionalization with natural polymers and biomaterials, as discussed above, have been shown to reduce potential toxic effects and improve therapeutic targeting, an in-vivo environment is required to observe neurotoxicity, drug efficacy, and immune responses. More study should focus on identifying the targeting properties of ligands for preclinical trials.

In this review, we have discussed the current treatment strategies along with the advances and contributions of biomaterial-coated magnetic nanoparticles along with recent breakthroughs in receptor-mediated and stimuli-responsive active targeted techniques in the therapeutic modalities of OS. The complex biology of osteosarcoma is still being vigorously studied and understood to improve prognosis, and quality of life, and to streamline efficient novel agents in advancing the treatment. Despite continual scientific advances, the management of complications remains critical, necessitating the development of rapid, accurate, and efficient methods of diagnosis and treatment for cure and survival while maintaining an exceptional quality of life. It is anticipated that multifunctional iron oxide nanosystems and their stimuli-responsive targeting techniques, with their distinct properties of increased stability and accelerated tumor accumulation, will be a potential material for biomedical applications in the coming years, and they may provide efficient solutions to current osteosarcoma-related problems. The present and future clinical studies will give optimism that an intervention can be developed. The scientific community's joint efforts, together with medical and industrial assistance, are critical in bringing improved treatment facilities to patients.

## Data Availability

No datasets were generated or analysed during the current study.

## Abbreviations

OS: Osteosarcoma
MNP: Magnetic nanoparticles
RT: Radiation therapy
RR: Radioresistance
SPIONS: Super paramagnetic iron oxide nanoparticles
TAA: Tumour associated antigens
PDT: Photo dynamic therapy
PTT: Photo thermal therapy
MHT: Magnetic hyperthermia
FDA: Food and drug administration
